# GmNF-YC4-2 Increases Protein, Exhibits Broad Disease Resistance and Expedites Maturity in Soybean

**DOI:** 10.3390/ijms22073586

**Published:** 2021-03-30

**Authors:** Seth O’Conner, Wenguang Zheng, Mingsheng Qi, Yuba Kandel, Robert Fuller, Steven A. Whitham, Ling Li

**Affiliations:** 1 Department of Biological Sciences, Mississippi State University, Mississippi State, MS 39762, USA; sjo88@msstate.edu (S.O.); robmfuller2@gmail.com (R.F.); 2 Department of Genetics, Development and Cell Biology, Iowa State University, Ames, IA 50011, USA; wgzheng2008@gmail.com; 3 Department of Plant Pathology and Microbiology, Iowa State University, Ames, IA 50011, USA; tonyqms@gmail.com (M.Q.); ykandel@iastate.edu (Y.K.); swhitham@iastate.edu (S.A.W.)

**Keywords:** NF-YC4 transcription factor, early maturation, disease resistance, seed protein

## Abstract

The NF-Y gene family is a highly conserved set of transcription factors. The functional transcription factor complex is made up of a trimer between NF-YA, NF-YB, and NF-YC proteins. While mammals typically have one gene for each subunit, plants often have multigene families for each subunit which contributes to a wide variety of combinations and functions. Soybean plants with an overexpression of a particular NF-YC isoform *GmNF-YC4-2* (Glyma.04g196200) in soybean cultivar Williams 82, had a lower amount of starch in its leaves, a higher amount of protein in its seeds, and increased broad disease resistance for bacterial, viral, and fungal infections in the field, similar to the effects of overexpression of its isoform *GmNF-YC4-1* (Glyma.06g169600). Interestingly, *GmNF-YC4-2-OE* (overexpression) plants also filled pods and senesced earlier, a novel trait not found in *GmNF-YC4-1-OE* plants. No yield difference was observed in *GmNF-YC4-2-OE* compared with the wild-type control. Sequence alignment of GmNF-YC4-2, GmNF-YC4-1 and AtNF-YC1 indicated that faster maturation may be a result of minor sequence differences in the terminal ends of the protein compared to the closely related isoforms.

## 1. Introduction

Effectively feeding an ever-growing global population begins with nutrient rich, resilient crops. Over one in seven people in the world do not have access to a sufficient protein supply in their diets [1]. In addition, the majority of the population relies on a plant-based diet for their protein uptake [2]. As most livestock also requires plant-based diets for their protein, the dietary needs of the world can mostly be attributed to plants. Thus, generating plants with increased protein content can help efficiently feed the global population.

Crop plants such as *Glycine max* (soybean) are grown in a wide range of environments and face a multitude of both biotic and abiotic challenges. While plants have evolved a complex immune system [3,4,5,6,7], crops across the world suffer yield loss due to diseases [8,9,10,11] and environmental factors [12,13]. Many genetic engineering methods have been used to combat these factors [8,14]. However, many of these induce constitutively active defense responses that can in turn negatively affect the growth and yield of the plant [15,16]. For example, silencing mitogen-activated protein kinase *MAPK4* in soybean plants severely stunts the plants when providing a resistance to pathogens [17]. Therefore, genetic engineered resistance that allows for an enhanced pathogen resistance while avoiding major growth defects is important.

While plants have evolved a complex immune system to deal with minor biotic and abiotic perturbations, they have also evolved a more drastic mechanism to deal with seasonal environmental changes. As plants are sessile organisms, rather than maintaining full function throughout a harsh season, they instead undergo programmed cell death called senescence [18,19,20]. This process allows for nutrient re-localization, such as nitrogen remobilization from leaves to seeds, and it is important for seed quality and nitrogen use efficiency [21,22,23,24]. With the looming threat of climate change and the growing global population, the challenge set before us is to improve crop nitrogen use efficiency in order to allow for less reliance on nitrate fertilizers [22]. It has been speculated that stimulating autophagy during stress response could improve crops’ resistance to the effects of climate change [22].

Previous work from our lab has identified an orphan gene, Qua Quine Starch (*QQS*), in *Arabidopsis thaliana* with a role in carbon and nitrogen allocation and disease resistance: QQS increased leaf and seed protein content, and increased plant resistance to pathogens, bacterium, virus, fungus and pests without impacting the plant growth and yield [25,26,27,28]; Nuclear Factor Y subunit C4 (NF-YC4) was its interacting partner [25,26,27,28] and had similar functions as QQS. Further work showed that homologs of *NF-YC4* in crop species confer similar effects [25,26]. In soybean, *GmNF-YC4-1-OE* (overexpression) plants demonstrate increased protein content and broad disease resistance, similar to the effect of *AtNF-YC4-OE* Arabidopsis plants [25,26,27,29]. Here, we show that another NF-YC4 isoform in soybean *GmNF-YC4-2*, when overexpressed, has increased protein content and broad disease resistance with no significant effect on seed yield. Unlike *GmNF-YC4-1*, *GmNF-YC4-2* has a role in plant maturity and senescence—faster pod filling and earlier senescence. This enhanced role of *GmNF-YC4-2* may likely be the result of small changes in the gene sequence near the terminal ends. Our results demonstrate that overexpressing *GmNF-YC4-2* increased carbon and nitrogen allocation to protein, increased broad disease resistance, and hastened plant maturation. 

## 2. Results

### 2.1. GmNF-YC4-2-OE Plants Have High Transcript Levels of GmNF-YC4-2 in Leaves and Seeds

**Figure 1:**
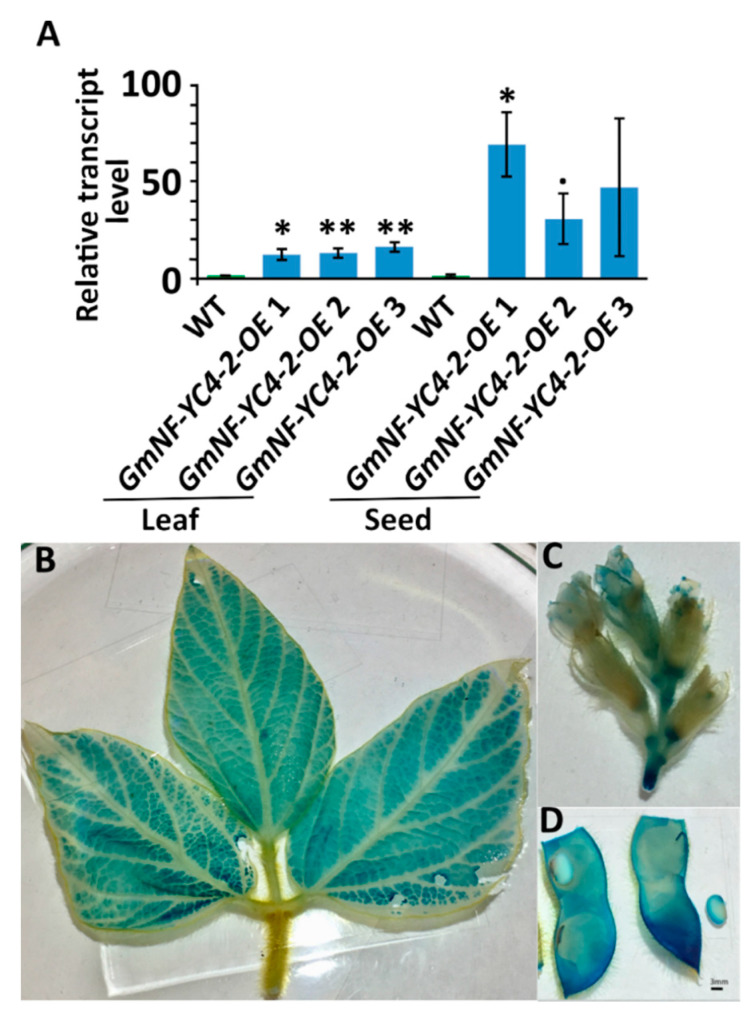
*GmNF-YC4-2* transcript level was increased in leaf and seed tissue in *GmNF-YC4-2-OE* plants. (**A**) Quantitative reverse transcription PCR of *GmNF-YC4-2* transcript level. GUS staining of 35S-QQS-GUS soybean plants shows 35S drove expression of GUS in leaves (**B**), flowers (**C**), and pods/seeds (**D**). All data in bar chart shows mean ± SE (Standard Error), *n* = 3. A two-tailed Student’s t-test was used to compare *GmNF-YC4-2-OE* and WT, ** *p* < 0.01; * *p* < 0.05; ^•^ *p* < 0.1.

To confirm the efficacy of our OE vectors, the transcript level of *GmNF-YC4-2* was assessed in leaves and seeds. The transcript level of *GmNF-YC4-2* in leaves of *GmNF-YC4-2-OE* lines ranged from 12.3-16.4 average relative quantification (RQ) compared to wild type (WT) (Figure 1A). That magnitude was increased in seeds where the average RQ ranged from 31.0-69.3 (Figure 1A). The validity of the 35S driven OE vectors used in this study was assessed by GUS (β-glucuronidase) staining of 35S-QQS-GUS constructs in soybean. Moderate to high levels of GUS signal were present in leaves, flowers, pods and seeds, indicating 35S activity in these tissues (Figure 1B–D) and confirming previous studies demonstrating no tissue specificity for the CaMV 35S promoter [30].

### 2.2. GmNF-YC4-2 Is Involved in Regulation of Plant Composition

**Figure 2:**
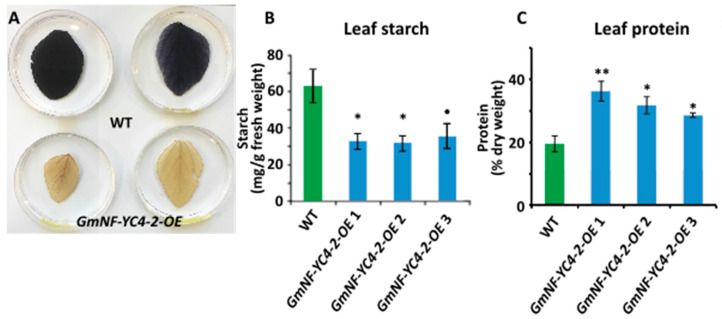
Leaf starch and protein composition was altered in *GmNF-YC4-2-OE* plants. Leaf starch content in *GmNF-YC4-2-OE* plants was decreased when compared to WT, indicated by starch staining (**A**) and quantification (**B**). (**C**) Leaf protein content was increased in *GmNF-YC4-2-OE* plants. All data in bar charts show mean ± SE, *n* = 3 or 4 (in **C**). A two-tailed Student’s t-test was used to compare *GmNF-YC4-2-OE* and WT, ** *p* < 0.01; * *p* < 0.05; ^•^ *p* < 0.1.

Similar to previous studies in *GmNF-YC4-1-OE* plants [27], *GmNF-YC4-2-OE* plants also showed altered leaf and seed composition. *GmNF-YC4-2-OE* plants had a decrease in leaf starch (Figure 2A,B, *p* < 0.05 for *GmNF-YC4-2-OE* 1,2 and *p* < 0.1 for *GmNF-YC4-2-OE* 3) and leaf protein content was increased (Figure 2C, *p* < 0.01 for *GmNF-YC4-2-OE* 1 and *p* < 0.05 for *GmNF-YC4-2-OE* 2,3).

**Figure 3:**
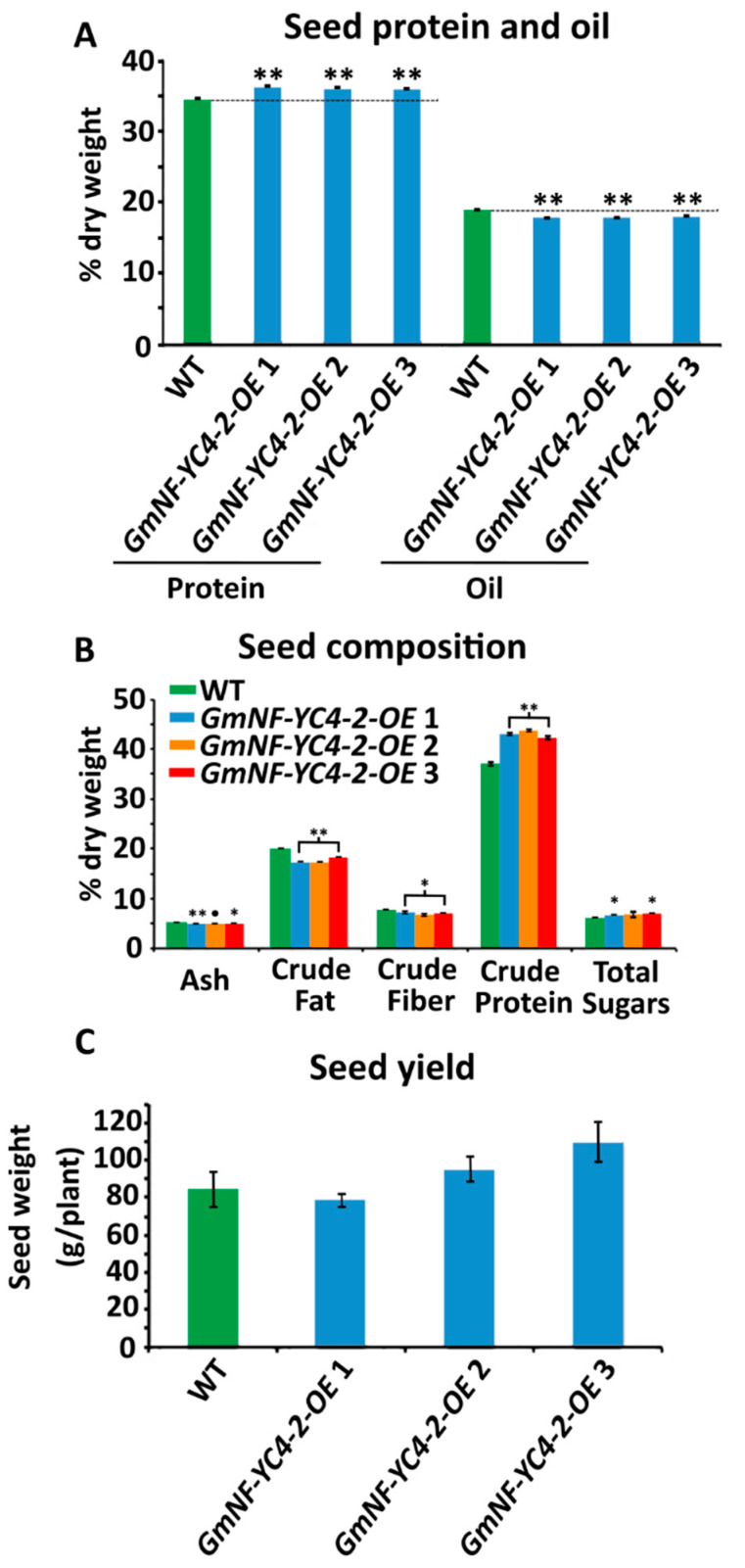
Seed composition was altered in *GmNF-YC4-2-OE* plants. (**A**) Seed protein content was significantly increased in all lines while oil content was significantly decreased. Composition was analyzed by near infrared spectroscopy (NIRS). (**B**) Ash content, crude fat and crude fiber were decreased compared to WT, while crude protein and total sugars were increased. Composition was analyzed by chemical methods. (**C**) No significant difference in seed yield was found. All data in bar charts show mean ± SE, (in **A**) *n* = 235 (WT), 125 (*GmNF-YC4-2-OE* 1), 155 (*GmNF-YC4-2-OE* 2), 81 (*GmNF-YC4-2-OE* 3), (in **B**) *n* = 3, (in **C**) *n* = 20 (WT), 24 (*GmNF-YC4-2-OE* 1), 17 (*GmNF-YC4-2-OE* 2), and 26 (*GmNF-YC4-2-OE* 3). A two-tailed Student’s t-test was used to compare *GmNF-YC4-2-OE* and WT; ** *p* < 0.01, * *p* < 0.05, ^•^ *p* < 0.1.

Metabolic analysis on *GmNF-YC4-2-OE* seeds was performed to determine if this metabolic effect in leaf tissue was unique. Near Infrared Spectroscopy (NIRS) analysis on seeds showed a significant increase in protein levels and decreased oil levels in three independent *GmNF-YC4-2-OE* lines compared to WT plants (Figure 3A, *p* < 0.01 for all three lines). Chemical analysis on crushed seeds was performed to test levels of ash, crude fat, crude fiber, crude protein, and total sugars. Of these metabolites, ash (*p* < 0.01, <0.1, and <0.05, respectively), crude fat (*p* < 0.01 for all), and crude fiber (*p* < 0.05 for all) showed a significant decrease in OE lines, while protein (*p* < 0.01 for all) and total sugar (*p* < 0.05 for *GmNF-YC4-2-OE* 1 and 3) levels were increased (Figure 3B). In addition, *GmNF-YC4-2-OE* plants had an even higher seed protein content, approximately 5–10%, when compared to *GmNF-YC4-1-OE* plants (Figure S1, *p* < 0.01). Interestingly, there was no significant difference in seed yield per plant between WT and OE plants (Figure 3C). Therefore, *GmNF-YC4-2-OE* plants display similar metabolic alterations in both leaf and seed tissue.

### 2.3. GmNF-YC4-2 Confers Broad Disease Resistance

**Figure 4:**
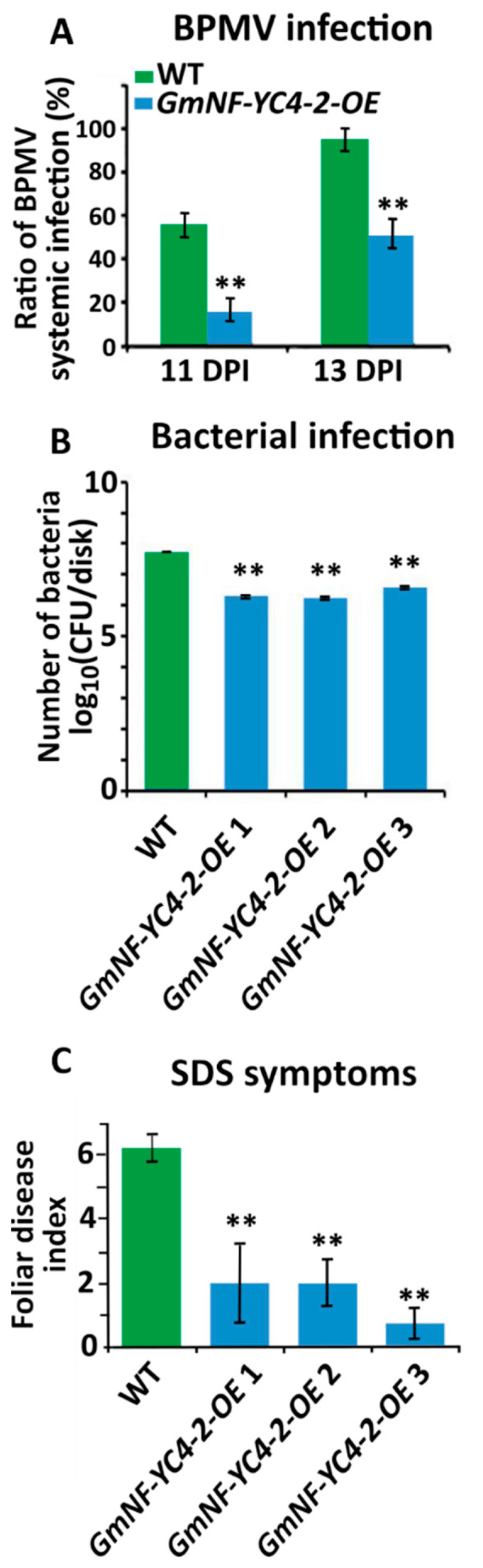
*GmNF-YC4-2-OE* plants showed enhanced disease resistance. (**A**) The BPMV viral foci rate was decreased at both 11 and 13 DPI in *GmNF-YC4-2-OE* plants compared to WT. (**B**) Growth of *Psg*R4 bacteria was reduced in *GmNF-YC4-2-OE* plants. CFU, colony forming units. (**C**) *GmNF-YC4-2-OE* plants also showed enhanced resistance to SDS. All data in bar charts show mean ± SE, (in **A** and **B**) *n* = 3, (in **C**) *n* = 5 (WT, *GmNF-YC4-2-OE* 1,2), 6 (*GmNF-YC4-2-OE* 3). A two-tailed Student’s t-test was used to compare *GmNF-YC4-2-OE* and WT; ** *p* < 0.01.

To test if *GmNF-YC4-2-OE* could affect interactions between soybean and pathogens, WT and transgenic plants were inoculated with a virus, bacterium, and fungus. *GmNF-YC4-2-OE* plants inoculated with a recombinant *Bean pod mottle virus* (BPMV) expressing green fluorescent protein (GFP) had reduced numbers of green fluorescent infection foci when compared to WT plants at both 11 and 13 days post inoculation (DPI) (Figure 4A, *p* < 0.01). The *GmNF-YC4-2-OE* plants were also infected with *Pseudomonas syringae* pv. *glycinea* Race 4 (*Psg*R4), the cause of bacterial blight. The growth of PsgR4 was decreased by 96.3%, 96.8%, and 92.8% in *GmNF-YC4-2-OE 1*, *GmNF-YC4-2-OE 2*, and *GmNF-YC4-2-OE 3* plants compared to Williams 82 control plants (Figure 4B, all *p* < 0.01).

The plants were planted in the field and inoculated with the fungus that leads to sudden death syndrome (SDS)-*Fusarium virguliforme*. OE plants also displayed less severe symptoms of SDS in a field inoculation trial with a 67.8%, 67.8%, and 88.1% decrease in foliar disease index for *GmNF-YC4-2-OE* 1, *GmNF-YC4-2-OE* 2, and *GmNF-YC4-2-OE* 3 respectively (Figure 4C, all *p* < 0.01). These data show that overexpression of *GmNF-YC4-2* in soybean plants confers enhanced disease resistance to the viral, bacterial, and fungal pathogens tested along with the altered leaf and seed composition.

### 2.4. GmNF-YC4-2 Regulates Plant Maturation

**Figure 5:**
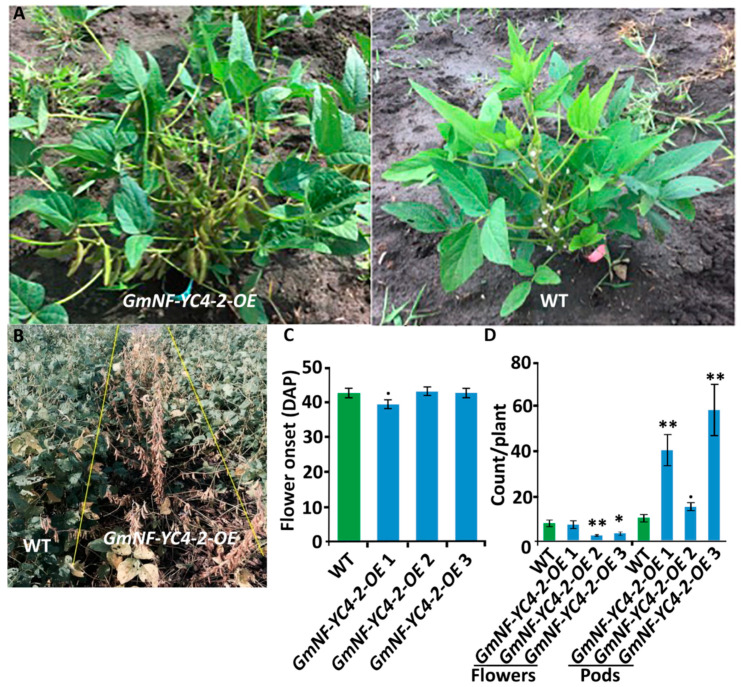
*GmNF-YC4-2-OE* plants transited from flowering stage to podding stage faster than WT plants. (**A**) *GmNF-YC4-2-OE* plants showed seeding pods while WT plants were still in the flowering stage at 77 DAP. (**B**) *GmNF-YC4-2-OE* plants senesced earlier than WT plants at 96 DAP. (**C**) The onset of flowering (DAP of first observed open flower) was slightly faster for *GmNF-YC4-2-OE* 1 plants but similar in *GmNF-YC4-2-OE* 2 and 3 plants compared to WT. (**D**) At 73 DAP, pod development for *GmNF-YC4-2-OE* plants was advanced compared to WT plants while the number of flowers was decreased. All data in bar charts show mean ± SE, (in **C**) *n* = 24 (WT), 46 (*GmNF-YC4-2-OE* 1), 39 (*GmNF-YC4-2-OE* 2), 26 (*GmNF-YC4-2-OE* 3); (in **D**) 15 (WT), 9 (*GmNF-YC4-2-OE* 1), 19 (*GmNF-YC4-2-OE* 2), 11 (*GmNF-YC4-2-OE* 3). A two-tailed Students t-test was used to compare *GmNF-YC4-2-OE* and WT; ** *p* < 0.01, * *p* < 0.05, ^•^ *p* < 0.1.

*GmNF-YC4-2-OE* plants displayed several aspects of expedited growth. In the field, *GmNF-YC4-2-OE* plants showed significantly faster pod development compared to the WT plants (Figure 5A). OE plants of all three lines also showed earlier senescence compared to WT (Figure 5B). As the date of flowering was similar for both WT and OE (Figure 5C), the faster maturation was seen mainly in the transition from flowering stage to pod development. *GmNF-YC4-2-OE* plants contained more pods than WT at 73 Days After Planting (DAP), while WT contained more flowers (Figure 5D, flowers: *p* < 0.01 for *GmNF-YC4-2-OE* 2, <0.05 for *GmNF-YC4-2-OE* 3; pods: *p* < 0.01 *GmNF-YC4-2-OE* 1 and *GmNF-YC4-2-OE* 3, <0.1 for *GmNF-YC4-2-OE* 2). Thus, *GmNF-YC4-2-OE* plants transited quickly from flowering to pod development and this likely impacted senescence independent of flowering time. OE plants fully matured around two weeks prior to WT plants.

### 2.5. Minor Sequence Differences in the Terminal Ends of GmNF-YC4-2 May Be Responsible for Early Maturation Phenotype

**Figure 6:**
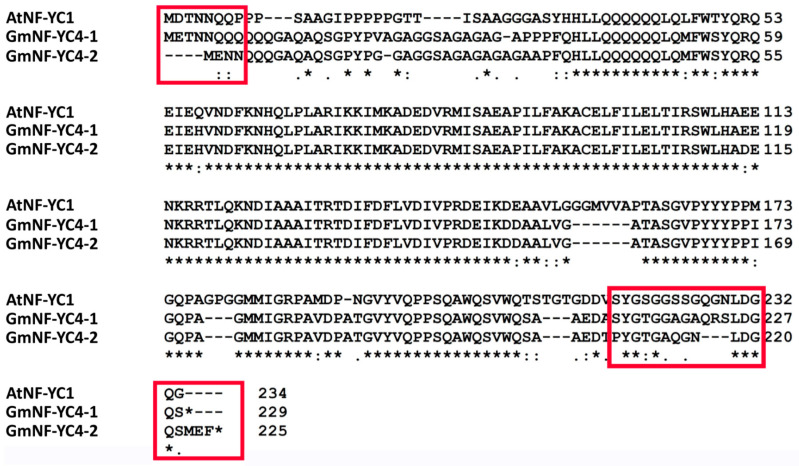
Protein sequence alignment of GmNF-YC4-2 with closest homolog in soybean (GmNF-YC4-1) and Arabidopsis (AtNF-YC1), neither of which has been shown to control maturation as we saw with GmNF-YC4-2. It is likely that sequence differences in the terminal ends (outlined in red) may be involved in GmNF-YC4-2′s function in early maturation. The ‘*’ at the end of the GmNF-YC4-1 and GmNF-YC4-2 sequences signifies a stop codon.

*GmNF-YC4-2* plays a similar role in carbon and nitrogen allocation and increasing broad disease resistance, as seen in both *AtNF-YC4* (an Arabidopsis isoform) and *GmNF-YC4-1*, which likely comes from the high sequence similarity within the proteins. However, unlike *GmNF-YC4-1*, *GmNF-YC4-2* causes faster maturation when overexpressed in soybeans. This novel function possibly comes from the small sequence dissimilarities near the terminal ends of the protein (Figure 6). A phylogenetic tree was estimated for multiple NF-YC family proteins in *A. thaliana*, *G. max* and *Zea mays*. Based on the tree, *GmNF-YC4-2* is more closely related to the NF-YC1 proteins of *A. thaliana* and *Z. mays* than to AtNF-YC4 (Figure S2). However, *GmNF-YC4-1* and *GmNF-YC4-2* are also the closest homologs in soybean to *AtNF-YC4*.

## 3. Discussion

### 3.1. GmNF-YC4-2-OE Plants Have High Transcript Levels of GmNF-YC4-2

We checked the relative transcript levels of *GmNF-YC4-2* in WT and *GmNF-YC4-2-OE* plants to determine the effectiveness of our OE vectors. The leaf tissue had a steady increase in *GmNF-YC4-2* expression, while the seeds had an overall greater magnitude of expression. There was also much more variation between samples (Figure 1A). GUS staining further verified the activity of 35S promoter in soybean leaves, flowers, and pods/seeds (Figure 1B–D). Together, these verified our *GmNF-YC4-2-OE* vectors and their effect on *GmNF-YC4-2* transcript level.

### 3.2. GmNF-YC4-2 and Nitrogen Metabolism and Pathogen Defense

We previously connected the *A. thaliana* orphan gene *Qua Quine Starch* (*QQS*) to a role in carbon and nitrogen allocation and subsequently found the same role in its interactor *AtNF-YC4* [25,28,31]. The closest NF-YC4 homologs in corn and soybean (*GmNF-YC4-1*) were also involved in regulating starch and protein partitioning [25,26]. Here, we demonstrated that another isoform of NF-YC4 in soybean, *GmNF-YC4-2*, is similarly involved in regulating starch and protein allocation (Figure 2 and Figure 3). In fact, *GmNF-YC4-2-OE* seeds had higher protein content than those from *GmNF-YC4-1-OE* plants (Figure S1). Oil content was also decreased in *GmNF-YC4-2-OE* seeds (Figure 3A). This is consistent with the known inverse relationship between soybean seed protein and seed oil [32]. *GmNF-YC4-2-OE* plants also demonstrated a similar phenotype to *GmNF-YC4-1-OE* plants [27] in response to bacterial, viral and fungal pathogens (Figure 4). These similarities are expected due to the high sequence similarity between both isoforms (Figure 6).

### 3.3. GmNF-YC4-2 and Plant Maturity

While the effect on metabolism and plant defense in *GmNF-YC4-2-OE* plants is similar to its isoform (*GmNF-YC4-1-OE* plants), a faster maturity phenotype was observed in *GmNF-YC4-2-OE* plants. In Arabidopsis, three NF-YC transcription factors—NF-YC3, 4, and 9—act in concert to regulate flower development [33]. Therefore, it is expected that some *G. max* NF-Y transcription factors may be important for plant development. *GmNF-YC4-2-OE* plants demonstrated several aspects of faster maturity. The onset of flowering was not significantly altered (Figure 5C), but the time from flowering to pod filling was hastened (Figure 5A,D). In addition, whole-plant senescence occurred earlier in *GmNF-YC4-2-OE* plants compared to WT in all three lines (Figure 5B). Typically, an alteration in plant senescence is paired with a change in flowering time. Here, the clear alteration in senescence appeared to be unlinked to a clear alteration in flowering time. In fact, a study in *A. thaliana* revealed a senescence quantitative trait loci (QTL) independent of flowering time [34]. In addition, QTLs for nitrogen use efficiency mapped back to the same region as the flowering independent QTL for senescence, leading the investigators to speculate a nitrogen metabolism gene to be responsible for flowering independent senescence [34]. In soybean, *GmNF-YC4-2* may be a gene that connects flowering independent senescence and nitrogen metabolism. Early maturing varieties of soybean can decrease the yield loss in late planted fields [35] and can allow for improved yields and limit crop loss in a two-crop planting system [36]. Thus, the *GmNF-YC4-2-OE* system may be used to improve either late-field planting or used in a two-crop system.

### 3.4. GmNF-YC4-2 Sequence Analysis

The sequences of *GmNF-YC4-1* and *GmNF-YC4-2* are more closely related to AtNF-YC1 than AtNF-YC4 (Figure 6 and Figure S2). However, based on the high similarities of function between AtNF-YC4 and the two *G. max* NF-YC4 homologs, they have been named NF-YC4 genes. In addition, these are the two closest homologs to AtNF-YC4 in soybean. As GmNF-YC4-1 and GmNF-YC4-2 have highly similar protein sequences, their overlap in function is expected. The novel function of *GmNF-YC4-2* in plant maturity is likely the result of small sequence differences found at the terminal ends (Figure 6). Based on this work, *GmNF-YC4-2* may be a highly active NF-YC family gene in plant metabolism (Figure 2 and Figure 3), disease resistance (Figure 4), and plant maturation (Figure 5).

## 4. Materials and Methods

### 4.1. Plant Materials

Soybean plants: the *GmNF-YC4-2* (Glyma.04g196200, in soybean genome version 1: Glyma04g37291) coding sequence overexpressing soybean lines in the Williams 82 background, expressed under the control of the constitutive cauliflower mosaic virus (CaMV) 35S promoter, were generated using the same vector and approach as described in [31]. Six lines of *GmNF-YC4-2-OE* plants were obtained after transformation. Of these six, all of them demonstrated the maturation phenotype and three lines were selected for further analysis. Similarly, 35S-QQS-GUS constructs were also generated with the same vector to overexpress QQS CDS-GUS. Transgenic plants were identified by both herbicide painting of leaf tissue and PCR to confirm T-DNA as in [31]. T3 generation seeds/plants were used for all analyses and segregated sibling WT plants were used as the controls. Quantitative reverse transcription PCR (RT-qPCR) was performed with GmNF-YC4 primers (F: 5′-CCTCCCAGGCATGGCAGTCC-3′, R: 5′-CCATCAAGGCTCCGCTGG-3′) and GmActin primers for endogenous control (F: 5′-GAGCTATGAATTGCCTGATGG-3′, R: 5′-CGTTTCATGAATTCCAGTAGC-3′). Relative expression was determined using the 2^-ΔΔCt^ method, as previously described [27].

GUS staining was performed as previously described using leaves (61 DAP), flowers (75 DAP) and pods (87 DAP) [28].

### 4.2. Disease Resistance Assays

The BPMV-GFP inoculation assay, Pseudomonas inoculation assay, and field SDS assay were performed as described in [27]. In brief, BPMV-GFP: soybean plants were grown for 14 days and then inoculated with BPMV-GFP. GFP foci were observed at 11 and 13 DPI (Days Post Inoculation), *n* = 5 with 6 plants per replicate.

Pseudomonas inoculation assay: the first trifoliate leaf on 23-day old soybeans was spray inoculated and bacterial levels were determined at 0 DPI and 4 DPI.

Field SDS assay: three independent lines were planted in six replicates in a randomized complete block design. The plants were planted in a field with a history of SDS and SDS inoculum was added to the soybeans at planting to induce uniform disease pressure. Fields were irrigated to create more favorable environment for the SDS. At stage R5 [37], the plants were assessed for SDS symptoms. SDS Incidence was estimated based on the number of plants with foliar symptoms in each row and SDS severity was estimated in the scale of 1-9 as previously described [38,39]. The incidence and severity were converted to a foliar disease index (FDX).

### 4.3. Flowering/Seeding Time

Soybean plants, of three lines (*GmNF-YC4-2-OE* 1, 2, and 3) from three independent transformation events of *GmNF-YC4-2-OE* and Williams 82 controls, were planted at Mississippi State University Research Farm on 03 May 2019. Beginning at the first signs of flowering, plants were visited daily to monitor the onset of flowering until flowers were present on every plant. At 73 DAP, during the transition from flowering to podding, flowers and pods were counted for each plant. *n* = 15 (WT), *n* = 9 (*GmNF-YC4-2-OE* 1), *n* = 19 (*GmNF-YC4-2-OE* 2), *n* = 11 (*GmNF-YC4-2-OE* 3).

### 4.4. Composition Analysis

The composition analysis was conducted as previously described [25,28,31], such as I_2_/KI staining for starch, starch quantification, and determination of protein content, *n* = 3 biological replicates.

Compositional analysis of soybean seeds (protein, oil, and fiber) was conducted with near-infrared spectroscopy (NIRS) using an Infratec 1229 whole grain analyzer. Biological replicates per independent line: *GmNF-YC4-2-OE* 1, *n* = 125; *GmNF-YC4-2-OE* 2, *n* = 155; *GmNF-YC4-2-OE* 3, *n* = 81; WT, *n* = 235.

Forty grams of seeds from each biological replicate (*n* = 3) in each of the three lines were crushed and sent to Eurofins for further composition testing of ash, crude fat, crude fiber, protein, and total sugars.

### 4.5. Seed Weight per Plant

Seeds were harvested from field-grown soybean plants and seed weight per plant was measured. Biological replicates per independent line: *GmNF-YC4-2-OE* 1, *n* = 24; *GmNF-YC4-2-OE* 2, *n* = 17; *GmNF-YC4-2-OE* 3, *n* = 26; and WT, *n* = 20.

### 4.6. Bioinformatics Analysis

Sequence analysis: for protein alignment visualization, sequences were aligned with Clustal Omega (1.2.4) multiple sequence alignment [40]. The maximum likelihood tree was created using MEGAX [41] based on the JTT+G+F model. The tree with the highest log likelihood was selected.

### 4.7. Accession Numbers

Sequence data from this article can be found under the following accession numbers in The Arabidopsis Genome Information Resource: QQS (At3g30720), NF-YC4 (At5g63470), NF-YC1 (At3g48590), and in LegumeIP: GmNF-YC4-1 (Glyma06g17780) and GmNF-YC4-2 (Glyma04g196200).

## Data Availability

Sequences for GmNF-YC4-1 and GmNF-YC4-2 were found at https://www.soybase.org/ (Access date 05 March 2021) and the sequence for AtNF-YC1 was found at https://www.arabidopsis.org/ (Access date 05 March 2021). All other sequences were found at https://www.ncbi.nlm.nih.gov/ (Access date 05 March 2021).

## Supplementary Materials

The following are available online at https://www.mdpi.com/article/10.3390/ijms22073586/s1. Figure S1: *GmNF-YC4-2-OE* lines had higher seed protein content than *GmNF-YC4-1-OE* lines. Figure S2: A maximum likelihood tree for the NF-YC family of proteins in *Arabidopsis thaliana*, *Glycine max*, and *Zea mays*.
