# Supplementary material for: GmNF-YC4-2 Increases Protein, Exhibits Broad Disease Resistance and Expedites Maturity in Soybean
Source: Int J Mol Sci. 2021 Mar 30;22(7):3586. doi: 10.3390/ijms22073586 (PMC8036377; doi:10.3390/ijms22073586)
Supplement: Supplementary file 1 [file ijms-22-03586-s001.pdf]

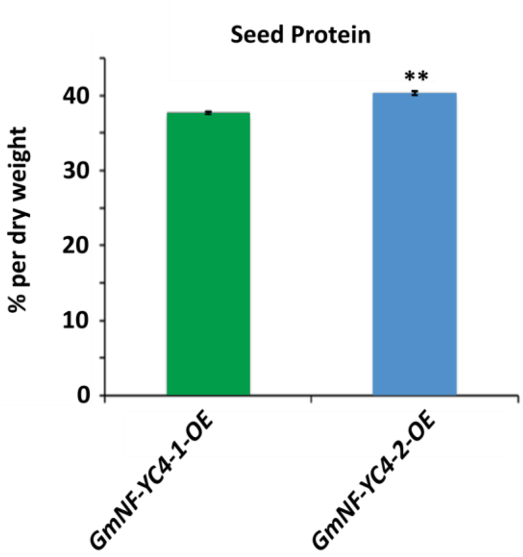

Figure S1. *GmNF-YC4-2-OE* lines had a higher seed protein content than *GmNF-YC4-1-OE* lines**.** Protein was measured by Kjeldahl method. Seeds from nine plants from three independent transformation events (three plants/event) were tested for each genotype (n = 9). All data in bar charts show mean ± SE (Standard Error). A two-tailed Student's t-test was used to compare *GmNF-YC4-1-OE* and *GmNF-YC4-2-OE*; $^{**}P < 0.01$.

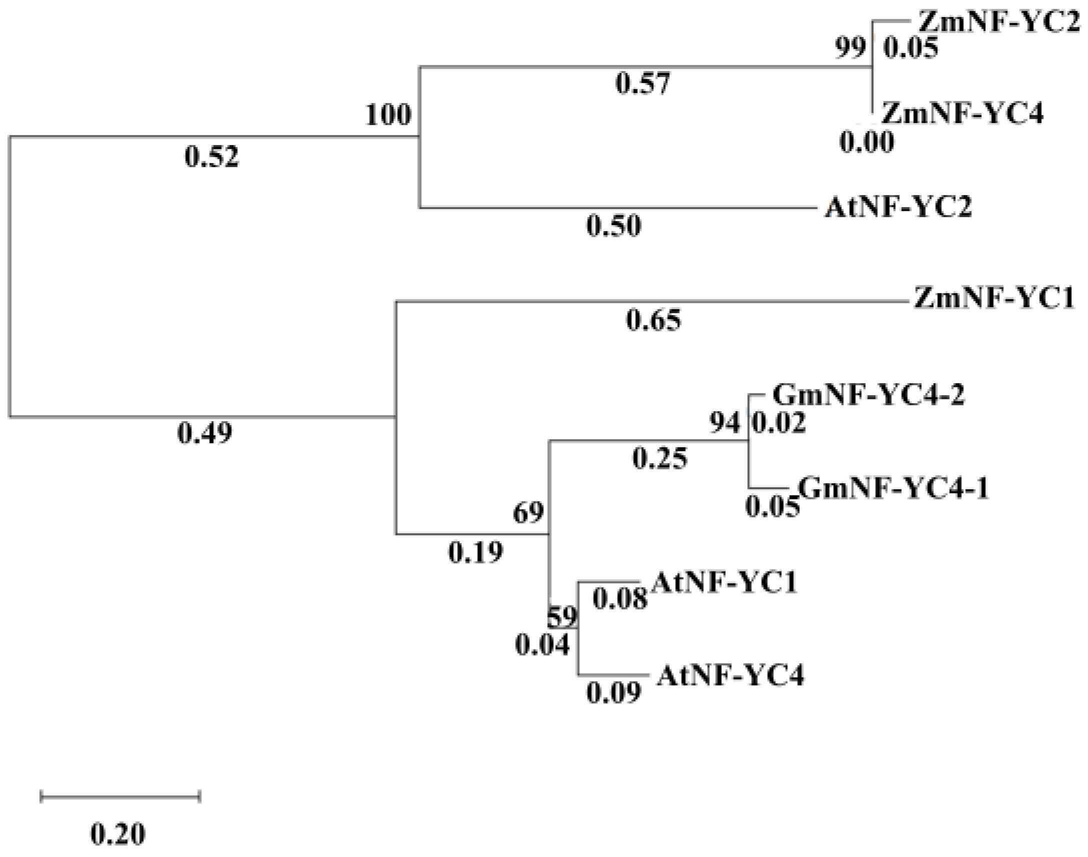

Figure S2. A maximum likelihood tree for the NF-YC family of proteins in *Arabidopsis thaliana*, *Glycine max*, and *Zea mays*. A blastn search was performed with GmNF-YC4-2 as the query and top matches from each organism were selected for the tree. Branch lengths are measured in the number of substitutions per site.
